# *trans*-Dichloridobis[diphen­yl(thio­phen-2-yl)phosphane-κ*P*]palladium(II)

**DOI:** 10.1107/S160053681201478X

**Published:** 2012-04-13

**Authors:** Andrew R. Burgoyne, Reinout Meijboom, Haleden Chiririwa, Leo Kirsten

**Affiliations:** a Research Centre for Synthesis and Catalysis, Department of Chemistry, University of Johannesburg, PO Box 524 Auckland Park, Johannesburg 2006, South Africa

## Abstract

The title compound, *trans*-[PdCl_2_(C_16_H_13_PS)_2_], forms a monomeric complex with a *trans*-square-planar geometry. The Pd—P bond lengths are 2.3387 (11) Å, as the Pd atom lies on an inversion point, while the Pd—Cl bond lengths are 2.2950 (12) Å.

## Related literature

For a review on related compounds, see: Spessard & Miessler (1996 ▶). For the synthesis of the starting materials, see: Drew & Doyle (1990 ▶). For (*R*_3_P)_2_PdCl_2_ compounds with consanguinities, see: Muller & Meijboom (2010 ▶); Meijboom (2011 ▶); Burgoyne *et al.* (2012 ▶); Ogutu & Meijboom, (2011 ▶). For their applications, see: Bedford *et al.* (2004 ▶).
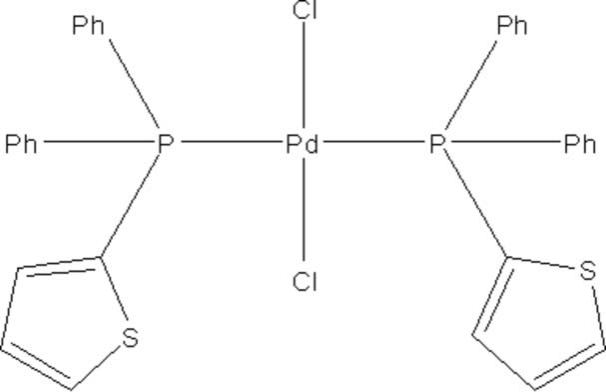


## Experimental

### 

#### Crystal data

[PdCl_2_(C_16_H_13_PS)_2_]
*M* *_r_* = 713.91
Monoclinic, 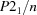

*a* = 9.019 (2) Å
*b* = 18.427 (4) Å
*c* = 9.658 (2) Å
β = 110.14 (4)°
*V* = 1507.0 (7) Å^3^
*Z* = 2
Mo *K*α radiation
μ = 1.06 mm^−1^
*T* = 100 K
0.20 × 0.20 × 0.16 mm

#### Data collection

Bruker X8 APEXII 4K KappaCCD diffractometer
Absorption correction: multi-scan (*SADABS*; Bruker, 2007 ▶) *T*_min_ = 0.812, *T*_max_ = 0.841
20792 measured reflections
3747 independent reflections
3591 reflections with *I* > 2σ(*I*)
*R*_int_ = 0.019

#### Refinement

*R*[*F* ^2^ > 2σ(*F* ^2^)] = 0.051
*wR*(*F* ^2^) = 0.111
*S* = 1.19
3747 reflections
198 parameters
40 restraints
H-atom parameters constrained
Δρ_max_ = 1.54 e Å^−3^
Δρ_min_ = −1.49 e Å^−3^

### 

Data collection: *APEX2* (Bruker, 2007 ▶); cell refinement: *SAINT-Plus* (Bruker, 2007 ▶); data reduction: *SAINT-Plus* and *XPREP* (Bruker, 2007 ▶); program(s) used to solve structure: *SHELXS97* (Sheldrick, 2008 ▶); program(s) used to refine structure: *SHELXL97* (Sheldrick, 2008 ▶); molecular graphics: *DIAMOND* (Brandenburg & Putz, 2005 ▶); software used to prepare material for publication: *WinGX* (Farrugia, 1999 ▶).

## Supplementary Material

Crystal structure: contains datablock(s) I, global. DOI: 10.1107/S160053681201478X/hb6729sup1.cif

Structure factors: contains datablock(s) I. DOI: 10.1107/S160053681201478X/hb6729Isup2.hkl

Additional supplementary materials:  crystallographic information; 3D view; checkCIF report

## Figures and Tables

**Fig. 1.**
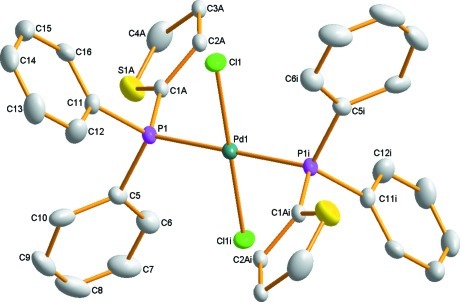
The structure of the trans-dichlorobis {diphenyl(thiophenyl-2-yl)phosphine}palladium(II) showing 50% probability displacement ellipsoids. Symmetry code to generate molecule through inversion point: - x, - y, 1 - z. Hydrogen atoms were omitted for clarity.

**Fig. 2.**
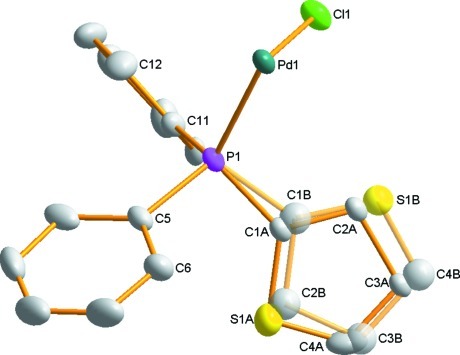
The structure of the disordered phenyl ring within trans-dichlorobis {diphenyl(thiophenyl-2-yl)phosphine}palladium(II), with the lower occupancy atoms shown as semi-transparent.

## supplementary crystallographic information

### Comment

The catalytic abilities of palladium metal centre complexes make them amongst the most popular catalytic precursors in organic synthesis. They are used in carbon-carbon bond formation reactions like the Heck, Stille and Suzuki reactions (Bedford *et al.*, 2004).

[PdCl_2_(*L*)_2_] (*L* = tertiary phosphine, arsine or stibine) complexes can conveniently be prepared by the substitution of 1,5-cyclooctadiene (COD) from [PdCl_2_(COD)]. The title compound, *trans*-[PdCl_2_{P(C_6_H_5_)_2_(C_4_SH_3_)}_2_], crystallizes with the Pd atom on a center of symmetry and each pair of equivalent ligands in a mutually *trans* orientation. The geometry is, therefore, slightly distorted square planar and the Pd atom is not displaced out of the coordinating atoms plane. All angles in the coordination polyhedron are close to the ideal value of 90°, with P—Pd—Cl = 87.50 (5)° and P—Pd—Cl^i^ = 92.50 (5)°. As required by the crystallographic symmetry, the P—Pd—P^i^ and Cl—Pd—Cl^i^ angles are 180°.

The title compound compares well with other closely related Pd(II) complexes from the literature containing two chloro and two tertiary phosphine ligands in a *trans* geometry (Muller & Meijboom, 2010). The title compound, having a Pd—Cl bond length of 2.2950 (12) Å and a Pd—P bond length of 2.3387 (11) Å, fits well into the typical range for complexes of this kind. Notably the title compound did not crystallize as a solvated complex; these type of Pd(II) complexes have a tendency to crystallize as solvates (Ogutu & Meijboom, 2011).

### Experimental

Dichloro(1,5-cyclooctadiene)palladium(II), [PdCl_2_(COD)], was prepared according to the literature procedure of Drew & Doyle (1990). A solution of diphenyl(thiophenyl-2-yl)phosphine (0.2 mmol) in dichloromethane (2.0 ml) was added to a solution of [PdCl_2_(COD)] (0.1 mmol) in dichloromethane (3.0 ml). Slow evaporation of the solvent gave the parent palladium compound. Recrystallization from dichloromethane afforded crystals of the title compound with 60% yield.

### Refinement

A disorder refinement model was applied to the thiophene ring. Ellipsoid displacement constraints (SIMU) were used to improve the model of the structure. The occupation parameters were linked to a free variable with a distribution of 0.57 (1):0.43 (1). P1, C1A, C2A and C3A were all constrained to have equal ADPs. All hydrogen atoms were positioned geometrically with C—H = 0.95 Å for aromatic H atoms. All hydrogen atoms were allowed to ride on their parent atoms with *U*_iso_(H) = 1.2*U*_eq_. The remaining highest electron peak was 1.54 at 0.06 Å from C1A and the deepest hole was -1.49 at 0.24 Å from C4B.

### Crystal data

**Table:**

[PdCl_2_(C_16_H_13_PS)_2_]	*F*(000) = 720.0
*M**_r_* = 713.91	*D*_x_ = 1.573 Mg m^−^^3^
Monoclinic, *P*2_1_/*n*	Mo *K*α radiation, λ = 0.71073 Å
Hall symbol: -P 2yn	Cell parameters from 9973 reflections
*a* = 9.019 (2) Å	θ = 2.5–28.3°
*b* = 18.427 (4) Å	µ = 1.06 mm^−^^1^
*c* = 9.658 (2) Å	*T* = 100 K
β = 110.14 (4)°	Cube, orange
*V* = 1507.0 (7) Å^3^	0.20 × 0.20 × 0.16 mm
*Z* = 2	

### Data collection

**Table:**

Bruker X8 APEXII 4K KappaCCD diffractometer	3747 independent reflections
Radiation source: fine-focus sealed tube	3591 reflections with *I* > 2σ(*I*)
Graphite monochromator	*R*_int_ = 0.019
φ and ω scans	θ_max_ = 28.4°, θ_min_ = 2.2°
Absorption correction: multi-scan (*SADABS*; Bruker, 2007)	*h* = −7→12
*T*_min_ = 0.812, *T*_max_ = 0.841	*k* = −24→24
20792 measured reflections	*l* = −12→12

### Refinement

**Table:**

Refinement on *F*^2^	Primary atom site location: structure-invariant direct methods
Least-squares matrix: full	Secondary atom site location: difference Fourier map
*R*[*F*^2^ > 2σ(*F*^2^)] = 0.051	Hydrogen site location: inferred from neighbouring sites
*wR*(*F*^2^) = 0.111	H-atom parameters constrained
*S* = 1.19	*w* = 1/[σ^2^(*F*_o_^2^) + (0.0193*P*)^2^ + 7.7351*P*] where *P* = (*F*_o_^2^ + 2*F*_c_^2^)/3
3747 reflections	(Δ/σ)_max_ < 0.001
198 parameters	Δρ_max_ = 1.54 e Å^−^^3^
40 restraints	Δρ_min_ = −1.49 e Å^−^^3^

### Special details

**Table:**

Experimental. The intensity data was collected on a Bruker X8 Apex II 4 K Kappa CCD diffractometer using an exposure time of 10 s/frame. A collection frame width of 0.5 ° covering up to θ = 28.4° resulted in 99% completeness accomplished.
Geometry. All e.s.d.'s (except the e.s.d. in the dihedral angle between two l.s. planes) are estimated using the full covariance matrix. The cell e.s.d.'s are taken into account individually in the estimation of e.s.d.'s in distances, angles and torsion angles; correlations between e.s.d.'s in cell parameters are only used when they are defined by crystal symmetry. An approximate (isotropic) treatment of cell e.s.d.'s is used for estimating e.s.d.'s involving l.s. planes.
Refinement. Refinement of *F*^2^ against ALL reflections. The weighted *R*-factor *wR* and goodness of fit *S* are based on *F*^2^, conventional *R*-factors *R* are based on *F*, with *F* set to zero for negative *F*^2^. The threshold expression of *F*^2^ > σ(*F*^2^) is used only for calculating *R*-factors(gt) *etc*. and is not relevant to the choice of reflections for refinement. *R*-factors based on *F*^2^ are statistically about twice as large as those based on *F*, and *R*- factors based on ALL data will be even larger.

### Fractional atomic coordinates and isotropic or equivalent isotropic displacement parameters (Å^2^)

**Table:**

	*x*	*y*	*z*	*U*_iso_*/*U*_eq_	Occ. (<1)
Pd1	0.5000	0.5000	0.5000	0.01794 (11)	
Cl1	0.70661 (12)	0.45351 (6)	0.69206 (10)	0.0280 (2)	
P1	0.52138 (11)	0.39844 (5)	0.36286 (10)	0.01804 (18)	
C5	0.4185 (5)	0.4000 (2)	0.1640 (4)	0.0225 (7)	
C11	0.7266 (4)	0.3790 (2)	0.3872 (4)	0.0205 (7)	
C10	0.5040 (6)	0.4028 (2)	0.0598 (5)	0.0310 (9)	
H10	0.6137	0.4036	0.0907	0.037*	
C16	0.7978 (5)	0.3108 (2)	0.4353 (4)	0.0231 (7)	
H16	0.7386	0.2724	0.4507	0.028*	
C12	0.8193 (6)	0.4352 (2)	0.3639 (5)	0.0325 (9)	
H12	0.7745	0.4806	0.3342	0.039*	
C13	0.9756 (5)	0.4240 (3)	0.3845 (5)	0.0362 (10)	
H13	1.0357	0.4612	0.3655	0.043*	
C6	0.2505 (5)	0.3988 (2)	0.1073 (5)	0.0309 (9)	
H6	0.1941	0.3982	0.1717	0.037*	
C7	0.1722 (7)	0.3984 (3)	−0.0409 (5)	0.0442 (12)	
H7	0.0624	0.3964	−0.0772	0.053*	
C8	0.2528 (7)	0.4010 (3)	−0.1360 (5)	0.0472 (14)	
H8	0.1975	0.4004	−0.2369	0.057*	
C9	0.4108 (7)	0.4042 (3)	−0.0871 (5)	0.0412 (12)	
H9	0.4608	0.4076	−0.1566	0.049*	
C15	0.9557 (5)	0.3024 (3)	0.4588 (5)	0.0340 (10)	
H15	1.0043	0.2581	0.4929	0.041*	
C14	1.0427 (5)	0.3582 (3)	0.4327 (5)	0.0347 (10)	
H14	1.1493	0.3512	0.4482	0.042*	
C1A	0.4428 (9)	0.3176 (4)	0.4104 (7)	0.01804 (18)	0.570 (6)
C2A	0.4540 (9)	0.3048 (5)	0.5578 (9)	0.01804 (18)	0.570 (6)
H2A	0.4988	0.3356	0.6377	0.022*	0.570 (6)
C3A	0.3748 (8)	0.2270 (4)	0.5617 (8)	0.0180 (6)	0.570 (6)
H3A	0.3626	0.2043	0.6432	0.022*	0.570 (6)
C4A	0.3287 (19)	0.2006 (7)	0.4190 (13)	0.041 (3)	0.570 (6)
H4A	0.2832	0.1550	0.3943	0.049*	0.570 (6)
S1A	0.3563 (4)	0.24996 (17)	0.3057 (3)	0.0386 (7)	0.570 (6)
C1B	0.4544 (10)	0.3199 (4)	0.4465 (6)	0.0178 (7)	0.430 (6)
C2B	0.3665 (17)	0.2577 (7)	0.3461 (10)	0.071 (6)	0.430 (6)
H2B	0.3453	0.2543	0.2451	0.085*	0.430 (6)
C3B	0.3197 (19)	0.2020 (6)	0.4418 (12)	0.030 (3)	0.430 (6)
H3B	0.2652	0.1589	0.4089	0.036*	0.430 (6)
C4B	0.3787 (12)	0.2298 (4)	0.6013 (8)	0.043 (2)	0.430 (6)
H4B	0.3662	0.2065	0.6820	0.052*	0.430 (6)
S1B	0.4619 (3)	0.30264 (15)	0.6042 (3)	0.0244 (7)	0.430 (6)

### Atomic displacement parameters (Å^2^)

**Table:**

	*U*^11^	*U*^22^	*U*^33^	*U*^12^	*U*^13^	*U*^23^
Pd1	0.01662 (18)	0.0228 (2)	0.01334 (18)	0.00304 (14)	0.00382 (13)	−0.00107 (14)
Cl1	0.0254 (5)	0.0336 (5)	0.0192 (4)	0.0079 (4)	0.0001 (3)	−0.0004 (4)
P1	0.0168 (4)	0.0231 (4)	0.0145 (4)	0.0022 (3)	0.0057 (3)	−0.0025 (3)
C5	0.0288 (19)	0.0210 (18)	0.0163 (16)	0.0016 (15)	0.0060 (14)	−0.0022 (13)
C11	0.0178 (16)	0.0247 (18)	0.0205 (16)	0.0008 (14)	0.0084 (13)	−0.0038 (14)
C10	0.040 (2)	0.0190 (18)	0.0249 (19)	0.0044 (17)	−0.0009 (17)	−0.0008 (15)
C16	0.0234 (18)	0.030 (2)	0.0170 (16)	−0.0005 (15)	0.0085 (14)	0.0036 (14)
C12	0.037 (2)	0.023 (2)	0.041 (2)	0.0011 (17)	0.0168 (19)	−0.0033 (17)
C13	0.029 (2)	0.040 (3)	0.044 (3)	−0.0133 (19)	0.019 (2)	−0.014 (2)
C6	0.032 (2)	0.034 (2)	0.0248 (19)	−0.0045 (18)	0.0067 (16)	−0.0041 (17)
C7	0.046 (3)	0.041 (3)	0.031 (2)	−0.011 (2)	−0.004 (2)	0.003 (2)
C8	0.071 (4)	0.035 (3)	0.025 (2)	−0.016 (2)	0.003 (2)	0.0041 (19)
C9	0.072 (4)	0.033 (2)	0.025 (2)	−0.004 (2)	0.025 (2)	−0.0026 (18)
C15	0.029 (2)	0.046 (3)	0.029 (2)	0.0137 (19)	0.0129 (17)	0.0069 (19)
C14	0.0185 (18)	0.058 (3)	0.029 (2)	0.0033 (19)	0.0089 (16)	−0.007 (2)
C1A	0.0168 (4)	0.0231 (4)	0.0145 (4)	0.0022 (3)	0.0057 (3)	−0.0025 (3)
C2A	0.0168 (4)	0.0231 (4)	0.0145 (4)	0.0022 (3)	0.0057 (3)	−0.0025 (3)
C3A	0.0168 (9)	0.0231 (5)	0.0145 (9)	0.0022 (8)	0.0057 (4)	−0.0025 (8)
C4A	0.036 (5)	0.028 (5)	0.058 (5)	−0.008 (4)	0.015 (5)	−0.015 (4)
S1A	0.0460 (14)	0.0398 (13)	0.0342 (13)	−0.0067 (10)	0.0191 (10)	0.0007 (9)
C1B	0.0166 (11)	0.0224 (11)	0.0145 (11)	0.0018 (10)	0.0056 (10)	−0.0026 (11)
C2B	0.067 (8)	0.077 (9)	0.062 (9)	0.025 (7)	0.014 (7)	−0.002 (8)
C3B	0.029 (5)	0.018 (5)	0.059 (6)	−0.007 (4)	0.037 (4)	−0.009 (5)
C4B	0.043 (4)	0.053 (4)	0.033 (4)	0.014 (4)	0.012 (4)	0.007 (4)
S1B	0.0279 (12)	0.0342 (13)	0.0112 (11)	−0.0045 (9)	0.0069 (10)	−0.0001 (10)

### Geometric parameters (Å, º)

**Table:**

Pd1—Cl1	2.2950 (12)	C7—H7	0.9300
Pd1—Cl1^i^	2.2950 (12)	C8—C9	1.340 (8)
Pd1—P1	2.3387 (11)	C8—H8	0.9300
Pd1—P1^i^	2.3387 (11)	C9—H9	0.9300
P1—C1A	1.777 (7)	C15—C14	1.370 (7)
P1—C11	1.820 (4)	C15—H15	0.9300
P1—C5	1.823 (4)	C14—H14	0.9300
P1—C1B	1.857 (6)	C1A—C2A	1.412 (9)
C5—C6	1.423 (6)	C1A—S1A	1.625 (7)
C5—C10	1.465 (6)	C2A—C3A	1.608 (10)
C11—C12	1.397 (6)	C2A—H2A	0.9300
C11—C16	1.414 (5)	C3A—C4A	1.384 (12)
C10—C9	1.378 (6)	C3A—H3A	0.9300
C10—H10	0.9300	C4A—S1A	1.507 (12)
C16—C15	1.371 (6)	C4A—H4A	0.9300
C16—H16	0.9300	C1B—S1B	1.534 (5)
C12—C13	1.369 (6)	C1B—C2B	1.534 (5)
C12—H12	0.9300	C2B—C3B	1.534 (5)
C13—C14	1.363 (7)	C2B—H2B	0.9300
C13—H13	0.9300	C3B—C4B	1.534 (5)
C6—C7	1.361 (6)	C3B—H3B	0.9300
C6—H6	0.9300	C4B—S1B	1.534 (5)
C7—C8	1.354 (8)	C4B—H4B	0.9300
			
Cl1—Pd1—Cl1^i^	180.000 (1)	C9—C8—C7	121.2 (5)
Cl1—Pd1—P1	87.52 (4)	C9—C8—H8	119.4
Cl1^i^—Pd1—P1	92.48 (4)	C7—C8—H8	119.4
Cl1—Pd1—P1^i^	92.48 (4)	C8—C9—C10	124.0 (5)
Cl1^i^—Pd1—P1^i^	87.52 (4)	C8—C9—H9	118.0
P1—Pd1—P1^i^	180.0	C10—C9—H9	118.0
C1A—P1—C11	106.3 (3)	C14—C15—C16	120.8 (4)
C1A—P1—C5	100.5 (2)	C14—C15—H15	119.6
C11—P1—C5	105.39 (18)	C16—C15—H15	119.6
C11—P1—C1B	105.0 (3)	C13—C14—C15	121.1 (4)
C5—P1—C1B	110.3 (2)	C13—C14—H14	119.5
C1A—P1—Pd1	114.0 (2)	C15—C14—H14	119.5
C11—P1—Pd1	111.29 (12)	C2A—C1A—S1A	110.6 (6)
C5—P1—Pd1	118.21 (13)	C2A—C1A—P1	120.3 (5)
C1B—P1—Pd1	105.9 (2)	S1A—C1A—P1	129.1 (4)
C6—C5—C10	118.6 (4)	C1A—C2A—C3A	107.1 (6)
C6—C5—P1	119.6 (3)	C1A—C2A—H2A	126.4
C10—C5—P1	121.8 (3)	C3A—C2A—H2A	126.4
C12—C11—C16	118.8 (4)	C4A—C3A—C2A	105.8 (7)
C12—C11—P1	118.1 (3)	C4A—C3A—H3A	127.1
C16—C11—P1	123.0 (3)	C2A—C3A—H3A	127.1
C9—C10—C5	115.5 (4)	C3A—C4A—S1A	116.4 (7)
C9—C10—H10	122.3	C3A—C4A—H4A	121.8
C5—C10—H10	122.3	S1A—C4A—H4A	121.8
C15—C16—C11	118.9 (4)	C4A—S1A—C1A	100.0 (5)
C15—C16—H16	120.5	S1B—C1B—C2B	108.0
C11—C16—H16	120.5	S1B—C1B—P1	133.1 (4)
C13—C12—C11	120.5 (4)	C2B—C1B—P1	118.8 (4)
C13—C12—H12	119.7	C3B—C2B—C1B	108.0
C11—C12—H12	119.7	C3B—C2B—H2B	126.0
C14—C13—C12	119.8 (4)	C1B—C2B—H2B	126.0
C14—C13—H13	120.1	C2B—C3B—C4B	108.0
C12—C13—H13	120.1	C2B—C3B—H3B	126.0
C7—C6—C5	120.2 (4)	C4B—C3B—H3B	126.0
C7—C6—H6	119.9	S1B—C4B—C3B	108.0
C5—C6—H6	119.9	S1B—C4B—H4B	126.0
C8—C7—C6	120.5 (5)	C3B—C4B—H4B	126.0
C8—C7—H7	119.8	C1B—S1B—C4B	108.0
C6—C7—H7	119.8		

Symmetry code: (i) −*x*+1, −*y*+1, −*z*+1.
